# Scoping implementation science for the beginner: locating yourself on the “subway line” of translational research

**DOI:** 10.1186/s12874-019-0783-z

**Published:** 2019-06-28

**Authors:** Meghan B. Lane-Fall, Geoffrey M. Curran, Rinad S. Beidas

**Affiliations:** 1 Department of Anesthesiology and Critical Care, University of Pennsylvania Perelman School of Medicine, Philadelphia, PA 19104 USA; 2 Penn Implementation Science Center at the Leonard Davis Institute of Health Economics (PISCE@LDI), University of Pennsylvania, Philadelphia, PA 19104 USA; 3 Penn Center for Perioperative Outcomes Research and Transformation, Philadelphia, PA 19104 USA; 4 Departments of Pharmacy Practice and Psychiatry, Center for Implementation Research, University of Arkansas for Medical Sciences, Central Arkansas Veterans Healthcare System, Little Rock, USA; 5 Department of Psychiatry, University of Pennsylvania Perelman School of Medicine, 3535 Market Street, 3rd floor, Philadelphia, PA 19104 USA; 6 Department of Medical Ethics and Health Policy, Perelman School of Medicine, University of Pennsylvania, Philadelphia, PA 19104 USA

**Keywords:** Implementation science, Translational research, Knowledge translation

## Abstract

**Background:**

Beginners to the discipline of implementation science often struggle to determine whether their research questions “count” as implementation science.

**Main text:**

In this paper, three implementation scientists share a heuristic tool to help investigators determine where their research questions fall in the translational research continuum. They use a “subway model” that envisions a journey to implementation research with stops along the way at efficacy and effectiveness research.

**Conclusions:**

A series of structured questions about intervention efficacy, effectiveness, and implementation can help guide researchers to select research questions and appropriate study designs along the spectrum of translational research.

## Introduction

Given evidence that it may take 17 years for research findings to be taken up into practice [1], there is a growing urgency in health services research to address the seemingly intractable research-to-practice gap. This urgency has fueled the development of implementation science, defined as the “scientific study of methods to promote the systematic uptake of research findings and other evidence-based practices into routine practice, and hence, to improve the quality and effectiveness of health services and care” [2]. The term “implementation science” is used in the United States, but this discipline is alternatively known as “dissemination and implementation research” and “knowledge translation” [3]. The growth of implementation science is evidenced by an increasing number of established frameworks [4] and recognized implementation outcomes [5]. We acknowledge that implementation science draws from and is related to numerous disciplines, including public health, psychology, organizational theory, human factors engineering, and others [2]. However, the similarities and differences between implementation science, dissemination and implementation research, knowledge translation, and other terms for the enterprise focused on facilitating the uptake of evidence into practice is beyond the scope of this commentary. Interested readers are referred to pre-existing literature addressing these distinctions [6, 7].

### Helping researchers distinguish between implementation science and related disciplines

**Fig. 1:**
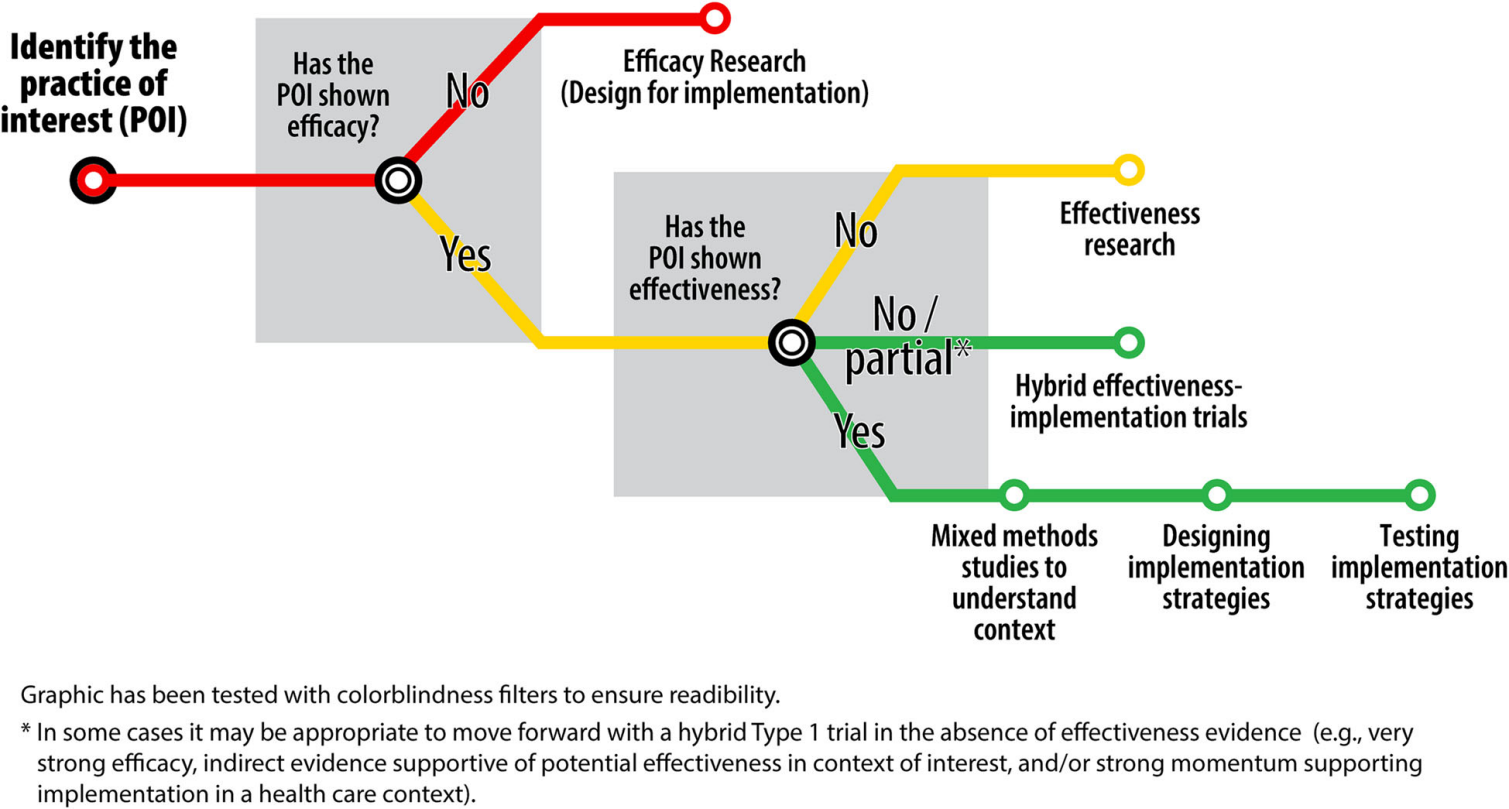
“Subway” schematic to guide researchers contemplating implementation studies of evidence-based interventions

There are growing efforts to build capacity for a cadre of implementation science researchers from both federal funders such as the National Institutes of Health [8] and academic universities [9]. We have been part of efforts to train researchers at our respective institutions and in regional, national, and international training efforts. These programs include conventional graduate-level courses, intensive 3-day immersion experiences, and informal and formal mentorship across a range of training stages including undergraduate, graduate, and postgraduate trainees. We engage trainees through a variety of mechanisms including both experiential and didactic approaches. Trainees across the training continuum (e.g., undergraduates to seasoned researchers making a lateral move into implementation science) often ask basic questions like the following: What kinds of research questions fall under the umbrella of implementation science? How do I know if my research focus is ready to be examined with an implementation science lens? How do I know when my intervention is ready for implementation? To answer these questions, we draw on a subway metaphor (Fig. 1), iteratively created with input from our trainees, that we have found to be a useful teaching aid. The goal of this brief commentary is to share our thinking and subway metaphor with the broader research community in the hopes that it can be useful.

We first encourage our trainees to identify the practice that they want to implement, a “practice of interest,” or POI. We use this terminology because there are myriad interventions, procedures, guidelines, tools, and practices that individuals or organizations might seek to implement [10]. Although our trainees tend to be interested in improving health care delivery and outcomes, we note that a “practice of interest” could refer to practices deployed in educational or community settings.

The next step is to evaluate the evidence in support of the POI. Although this step is second nature to implementation scientists, we find that other types of investigators may make their way to this discipline having identified an implementation strategy (e.g., audit and feedback, education) without having scrutinized the degree to which the practice of interest has a strong basis in evidence. Occasionally, students present an “evidence-based” concept or bundle - an example is the use of advanced care planning in patients with advanced illness [11] - whose particular manifestations or components have varying levels of evidence.

Therefore, the first branch point after identifying the practice of interest is to consider whether the POI has been shown to be efficacious. That is, does the POI improve clinical outcomes of interest when deployed under tightly controlled, ideally randomized, conditions? In the development of new drugs, this stage of research often occurs in Phase 2 or Phase 3 testing [12]. For non-drug interventions meant to improve health, efficacy testing may take many forms, including high-fidelity simulation or randomized controlled trials [13]. If the practice of interest has not yet shown efficacy, efficacy studies are needed. In this case, we encourage students to consider future implementation in the development and refinement of the intervention; we call this “designing for dissemination and implementation” [12].

If the POI has shown efficacy, the next question to consider is whether it has also shown effectiveness. We define effectiveness as “real world efficacy,” or evidence of benefit outside the realm of randomized controlled trials with strict inclusion and exclusion criteria [13]. Effectiveness studies should reflect real world practice with heterogeneous settings, clinicians, service providers, and patients or clients. If effectiveness studies showing benefit in the target population are lacking, we submit that two courses of action are reasonable. If no real world studies of the evidence-based practice have been conducted at all, these studies are needed to address the question of whether the POI works in the real world. Oftentimes however, effectiveness studies exist, but the setting or population of interest to the researcher is not adequately represented in the scientific literature. In this case, it may be prudent to test both effectiveness and implementation research questions using an effectiveness-implementation hybrid design [14]. For action-oriented researchers, this family of study designs is highly appealing because it allows simultaneous study of effectiveness and implementation outcomes, thereby expediting the translation of research findings into practice. However, these hybrid studies are often resource intensive and may be unfamiliar to readers and reviewers unfamiliar with implementation science. For these reasons, hybrid trials should only be undertaken by research teams with the expertise to design, execute, analyze, and disseminate them.

In the event that the POI has shown both efficacy and effectiveness, it is appropriate to proceed with studies focused on implementation [15]. Depending on what is already known about the implementation context and potential strategies, these studies might focus on contextual inquiry of implementation determinants (i.e., barriers and facilitators), the development and/or selection of implementation strategies [16, 17], and/or comparative effectiveness studies of different implementation strategies. In our experience, clinical audiences and some funders may demand effectiveness outcomes even in the context of strong effectiveness evidence. Later stage hybrid designs (Type 2 or Type 3) [14] represent one approach to focus on implementation while satisfying a desire to measure effectiveness outcomes. Irrespective of the implementation focus, mixed methods designs are commonly used in implementation research [18].

### Hypothetical case studies to test the implementation science schematic

In our color-coded schematic, only those arms in green (studies of implementation or hybrid studies) fall under the umbrella of “implementation science.” The following hypothetical research questions illustrate the utility of this schematic.

#### Research question 1

Does a new clinical protocol for sepsis improve patient outcomes? The POI is the clinical protocol. It has been pilot tested in a non-randomized fashion with promising results. Without randomized controlled trial data, we would say that evidence of efficacy is missing, so implementation studies are not yet warranted. The protocol should undergo efficacy testing, but the intervention developers should consider future implementation in the refinement and testing of the intervention (e.g., is it too complicated to work in routine clinical practice?).

#### Research question 2

Multiple randomized clinical trials have shown that Drug X improves outpatient blood pressure control in hypertensive patients. Does Drug X work in heterogeneous patient populations? The POI is drug X. Although there is evidence of efficacy, we do not yet know whether Drug X works in routine clinical practice. Efficacious interventions can fall flat in the real world once the realities of dosing, side effects, and interactions with other conditions and medications are considered. Drug X is not yet ready for studies of implementation, so effectiveness studies should occur next. However, effectiveness studies may yield observational data that will inform future implementation efforts.

#### Research question 3

Care coordination pathway Y improves outcomes for heart failure patients in both efficacy and effectiveness studies. Will it work for patients with diabetes? The POI is the care coordination pathway. It would be reasonable to study Pathway Y in effectiveness studies focused on patients with diabetes. Alternatively, a hybrid Type 1 trial would maintain a focus on effectiveness while either prospectively or retrospectively collecting information to inform future implementation efforts. Given that the existing effectiveness data arise from another population and that the intervention itself is complex and involves clinical process/workflow changes, a choice of a hybrid Type 1 study could be warranted. Relevant implementation outcomes for this early implementation evaluation include acceptability (i.e., how palatable or agreeable a POI is from the perspective of stakeholders), appropriateness (i.e., the perceived fit of the POI for a given setting, clinician, or patient), feasibility (i.e., the extent to which a POI can be successfully deployed in a given setting), and fidelity (i.e., the degree to which a POI is implemented as it was intended) [5]. These implementation outcomes and their relationship to more studied clinical effectiveness outcomes are explained in detail in seminal papers by Proctor and colleagues in 2009 [19] and 2011 [5].

#### Research question 4

Colon cancer screening leads to earlier cancer detection and improved patient outcomes [20]. What strategies can be used to increase colon cancer screening? The POI is colon cancer screening. This evidence-based practice is ripe for studies of implementation given the robust evidence base supporting it. Implementation studies are warranted. The focus of implementation studies will depend on what is known about the context to be studied. Potential study designs range from observational contextual inquiry to randomized controlled trials of implementation strategies.

## Discussion

We have outlined a series of structured questions useful in helping newcomers to implementation research answer the question, “Is this implementation science?” As with any heuristic tool, there are limitations. First, the schematic may also be difficult to apply prospectively to questions of de-implementation or in a retrospective fashion as with program evaluation. Further, the schematic places a great deal of emphasis on randomized controlled trials as the defining feature of research. Many research questions are not amenable to testing with randomized designs, either because the unit of analysis is an organizational unit (e.g., a hospital ward) or because the population or clinical locations to be studied do not accept randomized trials [21]. However, we have found this work useful in guiding discussions about implementation science research and knowledge translation and we hope that it is useful to others in both assisting in training efforts and also in allowing for evaluation of published research. It is likely that the schematic will be dynamic; we will continue to build upon it in the future. 

## Conclusions

A series of structured questions about intervention efficacy, effectiveness, and implementation can help guide researchers to select research questions and appropriate study designs along the spectrum of translational research.

## Data Availability

Data sharing is not applicable to this article as no datasets were generated or analyzed during the current study.

## Abbreviation

POI: Practice of interest
